# Particularities of Pyelonephritis With Multidrug-Resistant (MDR) Germs During Pregnancy: A Case-Control Study

**DOI:** 10.7759/cureus.71109

**Published:** 2024-10-08

**Authors:** Viorel Dragos Radu, Cristian Radu Costache, Pavel Onofrei, Rodica Radu, Bogdan Novac

**Affiliations:** 1 Department of Urology, Grigore T. Popa University of Medicine and Pharmacy, Iasi, ROU; 2 Department of Morphofunctional Sciences II, Grigore T. Popa University of Medicine and Pharmacy, Iasi, ROU; 3 Department of Urology, Elytis Hope Hospital, Iasi, ROU; 4 Department of Internal Medicine, Grigore T. Popa University of Medicine and Pharmacy, Iasi, ROU

**Keywords:** antibiotics, multidrug-resistant germs, pregnancy, pyelonephritis, risk factors

## Abstract

Introduction: The aim of this study was to analyze the characteristics of pyelonephritis with multidrug-resistant (MDR) bacteria in pregnant women in comparison to pyelonephritis with multidrug-sensitive bacteria in this particular patient group.

Methods: We conducted a retrospective study over a period of three years on a study group of 17 pregnant patients with pyelonephritis with MDR bacteria and on a control group of 52 pregnant patients with pyelonephritis with multidrug-sensitive bacteria. We analyzed the demographic data, the potential risk factors, aspects of the clinical picture, the incidence and type of bacteria involved, and their sensitivity spectrum.

Results: The patients in the study group had pyelonephritis on the right side (N=10, 58.83%), were in the second and third trimesters of pregnancy (N=16, 94.12%), and had a high percentage of anemia (N=9, 52.94%), as did the control group. Unlike the patients in the control group, the patients in the study group had a higher incidence of antibiotic treatment (N=17,100% vs. N=14, 29.92%, p<0.001), previous urological surgery, especially ureteral catheterization with insertion of double-J catheters (N=9, 52.94% vs. N=9, 17.30%, p=0.003), and an increased incidence of urosepsis on admission (N=11, 64.70% vs. N=11, 21.15%, p<0.001). *E. coli* (N=8, 47.05% vs. N=28, 53.85%, p=0.626) and *Klebsiella pn. *(N=5, 29.41% vs. N=12, 23.07%, p=0.598) were predominant in both study groups. Also, the hospitalization days were significantly higher in the study group compared with the control group (N=7.38±2,91 vs. N=5.02±1,87, p=0.002). The spectrum of microbial resistance was increased in favor of the study group for all antibiotics tested, with the exception of ampicillin, where resistance was increased in both groups. The Gram-negative bacteria responsible for the pyelonephritis in both groups retained 100% sensitivity to carbapenems and piperacillin/tazobactam.

Conclusions: Pregnant women in the second and third trimesters of pregnancy who have undergone right-sided double-J catheter insertion during pregnancy and/or who have received antibiotic treatment in the past are at increased risk for pyelonephritis with MDR. *E. coli* and *Klebsiella pn.,* bacteria are most commonly involved and maintain susceptibility to carbapenems and reserve penicillins.

## Introduction

Urinary tract infections in pregnant women, especially upper urinary tract infections, are a major health concern due to the potential impact on the mother and fetus [1-3]. At the same time, in recent years, the increasing incidence of urinary tract infections with multidrug-resistant (MDR) bacteria, hospital acquired, has become another public health problem, both in terms of morbidity and mortality [4], which, by its very nature, can also affect the special group of pregnant women.

There are numerous studies in the literature on pyelonephritis in pregnant women [5-7], with regard to the clinical picture, risk factors [8-10], evolution, and the pathogens involved, including some associated diseases such as gestational hydronephrosis or obstructive reno-ureteral lithiasis [11,12], as well as studies on the effects on the mother [13] and the fetus [2,14]. However, as pregnant women usually have minimal comorbidities and risk factors for MDR infection (hospitalization, surgical procedures, prolonged antibiotic treatment) [15-17], the potential risk of urinary tract infection with MDR bacteria, possibly pyelonephritis, is low. This is probably the reason why the studies reporting these cases are small [12,18], as to our knowledge there has been no European study presenting a series of such cases in the last 30 years.

For this reason, we have proposed to study this particular group of pregnant women with pyelonephritis with MDR bacteria with regard to the risk factors, the clinical picture, the bacteria involved, and their sensitivity spectrum.

## Materials and methods

Study design, data source, and study period

We conducted a retrospective case-control study of all pregnant patients hospitalized with acute pyelonephritis in the urology clinic of "Dr. C.I. Parhon" Hospital between 1.07.2021 and 30.06.2024. The study was approved by the Ethics Committee of the Hospital with no. 7183 on 13.08.2024. Patient data were extracted from the hospital's electronic registry, using the ICD-10 coding of the International Classification of Diseases, 10th edition [19]. All pregnant women with the code O09 (monitoring of a high-risk pregnancy) who were admitted to the hospital during this period were included. A total of 86 pregnant patients were identified.

**Figure 1:**
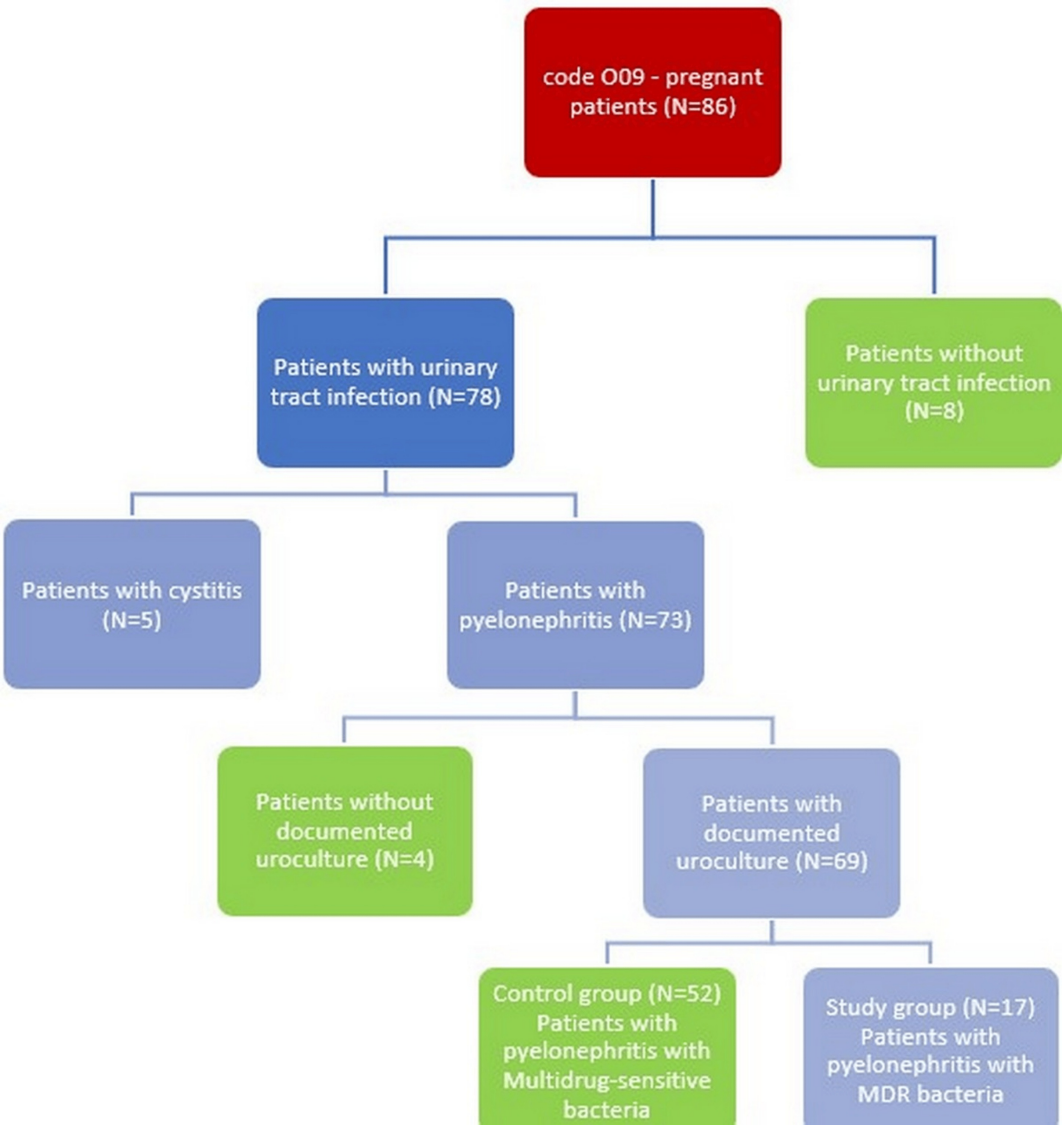
Selection of patients (flowchart) MDR: Multidrug-resistant

Of these, we excluded eight patients who did not present urinary infection, five patients who presented low urinary infection, and another four patients who did not have urine culture in the electronic registers. The result was 69 pregnant patients, hospitalized for pyelonephritis. This group of patients was divided into two groups depending on the presence or absence of MDR bacteria in the urine culture. The study group consisted of 17 patients with pyelonephritis with MDR bacteria, and the control group consisted of 52 patients with pyelonephritis with multidrug-sensitive bacteria. The flowchart of the selection of patients in the two groups is presented in Figure 1.

For the control group, we included all patients with pyelonephritis with multidrug-sensitive bacteria, creating a larger control group in order to increase the power of the statistical tests.

The diagnosis of pyelonephritis for the two study groups was based on the presence of clinical and paraclinical signs of upper urinary tract infection (febrile syndrome, lumbar pain, leukocytosis) and a positive urine culture. We defined a positive culture in urine samples with more than 100000 CFU/ml bacterial growth. Urine for uroculture was collected in the first hours after admission and before the start of empirical antibiotic treatment. Antimicrobial susceptibility testing was performed using the Kirby Bauer diffusimetric method, using the EUCAST clinical breakpoint tables valid at the time of strain isolation (v.11.0 for 2021, v.12.0 for 2022, v.13.0 for 2023 and 14.0 for 2024) for the interpretation of minimal inhibitory concentrations and inhibition zone diameters [20]. The following antibiotic discs were used: ampicillin (10 µg); amoxicillin-clavulanic acid (20-10 µg); piperacillin-tazobactam (30-6 µg); ceftazidime (10 µg); cefuroxime (30 µg); ceftriaxone(30micrograms); imipenem (10 µg); meropenem (10 µg); ciprofloxacin (5 µg); levofloxacin (5 µg); amikacin (30 µg); gentamicin (10 µg); nitrofurantoin (100 µg); trimethoprim-sulfamethoxazole (1.25-23.75 µg).

Isolates were classified as MDR if isolates were non-susceptible to at least one agent from more than three antimicrobial classes [21]. The diagnosis of urosepsis was based on The Third International Consensus Definitions for Sepsis and Septic Shock (Sepsis-3) [22].

Variables

We analyzed patient demographics such as age, site of infection, trimester of pregnancy, site of origin, and parity and comorbidities such as anemia, diabetes mellitus, and coexisting urologic disease. We used the term anemia for a hemoglobin level < 11mg/dl. We considered the first trimester of pregnancy from 1-12 weeks of gestation, the second trimester of pregnancy between 13 and 26 weeks of gestation, and the third trimester of pregnancy between 27 and 42 weeks of gestation. We analyzed the presence of risk factors for the development of MDR bacteria and for adverse outcomes, such as previous antibiotic treatment, previous urological surgery, and the type of surgery coexisting at the time of hospitalization for urinary tract obstruction, i.e. hydronephrosis. Regarding the clinical and paraclinical picture, we analyzed the presence of fever, leukocytosis, CRP, creatinine, the presence of urosepsis on admission, and the number of days of hospitalization. With regard to the bacteria involved, we analyzed their incidence and type as well as the sensitivity spectrum of the bacteria in the two groups.

Data analysis

Quantitative data were described using the mean and standard deviation. Qualitative data were described as percentages. The comparison between the two groups was performed using a non-parametric test for the comparison of means, the Mann-Whitney U test. The chi-square test and the Fisher exact test were used for comparing the percentages. We used the non-parametric test due to the relatively small size of the study group. We classified the differences as statistically significant when p <0.05, using IBM SPSS Statistics for Windows, Version 24 (Released 2016; IBM Corp., Armonk, New York, United States).

## Results

During the three-year study period, there were 17 pregnant women with acute pyelonephritis with MDR bacteria and 52 patients with pyelonephritis with multidrug-sensitive bacteria. The characteristics and demographic data of the two groups are shown in Table 1.

**Table 1:** The characteristics and demographic data of the two groups *The Fisher's exact test statistic value; ** Mann-Whitney U Test; *** Z score for Mann-Whitney U Test; the p-value is considered statistically significant for p<0.05 DM: Diabetes mellitus

		Study group (n=17)	Control group (n=52)	p (Chi-square test)	Chi-square value for the Chi-square test
Age (mean ± SD)		25.47±3.71	25.73±6.79	0.711**	0.369***
Infection site (N, %)	Right	10 (58.83%)	34 (65.38%)	0.625	0.238
	Left	3 (17.65%)	13 (25%)	Not significant at p<0.05	0.743*
	Bilateral	4 (23.52%)	5 (9.62%)	Not significant at p<0.05	0.208*
Trimester of pregnancy (N, %)	1st	1 (5.88%)	4 (7.69%)	Not significant at p<0.05	1*
	2nd	7 (41.18%)	29 (55.77%)	0.295	1.093
	3rd	9 (52.94%)	19 (36.53%)	0.231	1.429
Place of origin (N, %)	Rural	8 (47.06%)	28 (53.85%)	0.626	0.236
	Urban	9 (52.94%)	24 (46.15%)	0.626	0.236
Parity (N, %)	Nullipara	10 (58.82%)	34 (65.38%)	0.625	0.238
	Multipara	7 (41.18%)	18 (34.61%)	0.625	0.238
Comorbidities (N, %)	DM	1 (5.88%)	0 (0%)	Not significant at p<0.05	0.246*
	Anemia	9 (52.94%)	24 (46.15%)	0.724	0.123

There were no statistically significant differences between the two groups with regard to the age of the patients, the localization of the infection on the left and right, the time of diagnosis, the place of origin, and the presence of anemia. In both groups, the average age of the patients was over 25 years, the pyelonephritis was predominantly on the right side (N=10, 58.83% vs. N=34, 65.38%, p=0.625) and occurred predominantly in the second and third trimesters of pregnancy. There were also no differences between the two groups in terms of place of origin (urban: N=9, 52.94% vs. N=24, 46.15%, p=0.236) and parity (nulliparous: N=10, 58.82% vs. N=34, 65.38%, p=0.238). We note that a high percentage of women have anemia, with no differences between the two groups (N=9, 52.94% vs. N=24, 46.14%, p=0.236).

The results regarding potential risk factors, types of urological surgery history, and the presence of hydronephrosis at the time of diagnosis are shown in Table 2.

**Table 2:** Potential risk factors in the two groups *Chi-square test; **Chi-square value for the Chi-square test; the p-value is considered statistically significant for p<0.05

		Study group (n=17)	Control group (n=52)	p (Fisher's exact test)<0.05	Fisher's exact test statistic value
Previous antibiotic treatment (past 180 days) (N, %)		17 (100%)	14 (26.92%)	Significant at p<0.05	<0.001
Previous surgical procedures (N, %)		13 (76.47%)	11 (21.15%)	Significant at p<0.05	<0.001
Urologic surgical procedures (N, %)	Double-J catheter insertion	9 (52.94%)	9 (17.30%)	0.003*	8.436**
	Double-J catheter replacement	2 (11.76%)	2 (3.85%)	Not significant at p<0.05	0.252
	Nephrostomy catheter insertion	2 (11.76%)	0 (0%)	Not significant at p<0.05	0.058
	No surgical procedure	4 (23.52%)	37 (71.15%)	Significant at p<0.05	<0.001
Associated hydronephrosis (N, %)		15 (88.23%)	46 (88.46%)	Not significant at p<0.05	1

There was a statistically significant difference in terms of previous antibiotic treatment (N=17, 100% vs. N=14, 26.92%, p<0.001) and previous urological procedures, due to insertion of double-J catheters (N=9, 52.94% vs. N=9, 17.30%, p=0.003). In contrast, there were no differences in associated hydronephrosis, which occurred in over 88% of both groups.

The clinical and paraclinical data as well as the number of days of hospitalization are shown in Table 3.

**Table 3:** Clinical and paraclinical data of the two groups *The Fisher's exact test statistic value; **Chi-square test; ***Chi-square value for the Chi-square test; the p-value is considered statistically significant for p<0.05

	Study group (n=17)	Control group (n=52)	p (Mann-Whitney U Test)	Z score for the Mann-Whitney U Test
Fever (N, %)	16 (94.11%)	36 (69.29%)	Not significant at p<0.05	0.051*
Fever > 38°C (N, %)	11 (64.70%)	27 (51.92%)	0.357**	0.846***
Sepsis on admission (N, %)	11 (64.70%)	11 (21.15%)	<0.001**	11.189***
Leukocytosis (mean ± SD)	16213.53 (±2409.22)	16256 (±5568.43)	0.561	-0.577
CRP (mean ± SD)	107.59 (±77.93)	100.61 (±80.18)	0.689	-0.403
Creatinine (mean ± SD)	0.73 (±0.25)	0.67 (0.55)	0.036	-2.067
Hospital stay (days no. ± SD)	7.38 (±2.91)	5.02 (±1.87)	0.002	-2.992

There was no statistically significant difference between the two groups in terms of the presence and intensity of the inflammatory syndrome, fever (p=0.051), leukocytosis (p=0.0561), and CRP level (p=0.689). Instead, patients in the study group had a higher incidence of sepsis associated with pyelonephritis on admission (N=11, 64.70% vs. N=11, 21.15%, p<0.001), a higher number of days of hospitalization (p=0.002), and a higher mean creatinine (p=0.036).

The incidence and type of bacteria involved are shown in Table 4.

**Table 4:** The incidence and type of bacteria in the two groups * Chi square test; **Chi square value for Chi square test; the p-value is considered statistically significant for p<0.05

Type of bacteria	Study group (n=17)	Control group (n=52)	p (Fisher's exact test) <0.05	Fisher's exact test statistic value
E. coli (N, %)	8 (47.05%)	28 (53.85%)	0.626*	0.236**
Klebsiella pn. (N, %)	5 (29.41%)	12 (23.07%)	0.598*	0.276**
P. mirabilis (N, %)	0 (0%)	2 (3.85%)	Not significant at p<0.05	1
P. aeruginosa (N, %)	0 (0%)	2 (3.85%)	Not significant at p<0.05	1
Serratia marcescens (N, %)	1 (5.88%)	1 (1.92%)	Not significant at p<0.05	0.434
S. aureus (N, %)	3 (17.65%)	2 (3.85%)	Not significant at p<0.05	0.091
Enterococcus spp. (N, %)	0 (0%)	5 (9.61%)	Not significant at p<0.05	0.323

The incidence and type of bacteria in the two groups were similar, without statistically significant differences. In both groups, E. Coli and Klebsiella pn. were the most frequently encountered bacteria.

The susceptibility of the Gram-negative bacilli from the two groups to the antibiotics tested is shown in Table 5.

**Table 5:** Susceptibility to antibiotics of the Gram-negative bacilli in the two groups * Chi square test; **Chi square value for Chi square test; p-value is considered statistical significant for p<0.05

Type of tested antibiotic	Study group (n=14)	Control group (n=45)	p (Fisher's exact test) <0.05	Fisher's exact test statistic value
Ampicillin (N, %)	0 (0%)	2 (4.44%)	Not significant at p<0.05	1
Amoxicillin/acid clavulanic (N, %)	3 (21.42%)	30 (66.66%)	Significant at p<0.05	0.0048
Trimethoprim/sulfamethoxazole (N, %)	2 (14.28%)	37 (82.22%)	Significant at p<0.05	<0.001
Nitrofurantoin (N, %)	10 (71.43%)	42 (93.33%)	Significant at p<0.05	0.047
Ciprofloxacin (N, %)	7 (50%)	38 (84.44%)	0.008*	6.999**
Levofloxacin (N, %)	7 (50%)	38 (84.44%)	0.008*	6.999**
Cefuroxime (N, %)	4 (28.57%)	41 (91.11%)	Significant at p<0.05	0
Ceftriaxone (N, %)	4 (28.57%)	45 (100%)	Significant at p<0.05	0
Ceftazidime (N, %)	4 (28.57%)	45 (100%)	Significant at p<0.05	0
Piperacillin/Tazobactam (N, %)	14 (100%)	45 (100%)	Significant at p<0.05	1
Imipenem (N, %)	14 (100%)	45 (100%)	Significant at p<0.05	1
Meropenem (N, %)	14 (100%)	45 (100%)	Significant at p<0.05	1
Gentamicin (N, %)	3 (21.42%)	44 (97.77%)	Significant at p<0.05	<0.001

There was a statistically significant difference between the two groups with regard to susceptibility to amoxicillin/clavulanic acid, nitrofurantoin, ciprofloxacin, levofloxacin, cefuroxime, and gentamicin in terms of increased resistance of MDR bacteria (p<0.001). Resistance to ampicillin was increased in both groups. In both groups, there was 100% sensitivity to piperacillin/tazobactam, imipenem, and meropenem.

As far as pyelonephritis with S. aureus is concerned, there was resistance to cefuroxime, ceftriaxone, oxacillin, and doxycycline in three cases in the study group and resistance to cefepime, erythromycin, and trimethoprim/sulfamethoxazole in two cases. In the control group, there was resistance to doxycycline in one of the two cases. We did not consider resistance to enterococci, as there was no case in the study group.

## Discussion

Pyelonephritis with MDR bacteria in pregnant women occurs mainly in the third and second trimesters of pregnancy, following antibiotic treatment and/or urological procedures, especially after the insertion and replacement of double-J catheters, which are used for obstructive pyelonephritis, predominantly on the right side. The bacteria involved are predominantly E. coli and Klebsiella pn., and antibiotic resistance, although belonging to the MDR bacteria, does not extend to carbapenem antibiotics, but especially to antibiotics commonly used in pregnancy for the treatment of urinary tract infections, such as cefuroxime and amoxicillin/clavulanic acid.

When analyzing the results of the characteristic and demographic data, we note that the patients in both groups were relatively young with an average age of just over 25 years [8,9], the localization was predominantly on the right side and the infections occurred in the third and second trimesters of pregnancy, in contrast to other studies that reported an increased incidence only in the second trimester [2,5]. This can be explained by the association of gestational hydronephrosis, which occurs predominantly on the right side [23] and is more pronounced in the second and third trimesters of pregnancy [24]. In the study group, infection with MDR bacteria occurred less in the second trimester and more frequently in the third trimester, as these were due to some urological procedures and previous antibiotic treatments during pregnancy.

The infection occurred in both nulliparas and multiparas, in contrast to other studies that reported an increased incidence in nulliparas [5,9] and also in rural and urban patients, suggesting that they probably do not play an important role in the occurrence of pyelonephritis in pregnant women, even with MDR bacteria. We note that in both groups, as in other studies [1], there is a high percentage of anemia, but some comorbidities are absent, which is to be expected in young patients in the third decade of life.

When analyzing the possible risk factors for the occurrence of MDR pyelonephritis, we note a clear difference from the control group, namely the fact that all pregnant women were treated with antibiotics before the occurrence of MDR infection and most of them were hospitalized and underwent urological surgery, most of them insertion or replacement of double-J catheters. It is known that patients with long-term double-J catheters are at risk of developing pyelonephritis, also known as reflux pyelonephritis [4,25], even if some studies consider this procedure to be safe [26]. Although some studies have shown that the presence of double-J catheters and nephrostomy catheters in pregnant women reduces the rate of new urinary tract infections, including pyelonephritis [11], we have shown the opposite. Most patients with pyelonephritis with MDR bacteria in our study were carriers of double-J catheters and nephrostomies. In addition, we had no patients with open urological surgery, which can be explained by the youth of the patients and the fact that they are generally postponed until after birth if they are not urgent. However, in other local studies, postoperative urinary tract infections with MDR bacteria were not reported in pregnant women who underwent open surgery [27,28]. In the control group, most patients were diagnosed with pyelonephritis at first hospitalization, without prior antibiotic treatment. These results suggest that the risk factors for the occurrence of pyelonephritis with MDR bacteria in pregnant women are similar to the risk factors for the occurrence of urinary tract infections with MDR bacteria in the general population [29-32]. Therefore, the correct treatment of urinary tract infections in pregnancy, including cystitis [33], and the monitoring of pregnant women, especially in the second and third trimesters, for the occurrence of gestational hydronephrosis should be given utmost importance.

With regard to the clinical picture, we note that patients with pyelonephritis with MDR bacteria did not show a more pronounced inflammatory syndrome, leukocytosis, and CRP values being similar in the two study groups. However, more patients in the study group had urosepsis and fever above 38° C, indicating a possibly greater aggressiveness of the MDR bacteria and, in the case of this particular group of patients, perhaps explaining the longer duration of hospitalization in this group [3]. The serum creatinine level was also higher in the study group, which is probably due to the renal damage in sepsis.

As in the control group, E. coli and Klebsiella pn., the bacteria most frequently involved in the occurrence of urinary tract infections in pregnant women [2,5,7] and reported in other local studies to be the most common MDR bacteria in a urology clinic [30], predominated. However, we note the presence of other Gram-negative bacteria from the Enterobacterales group as well as S. aureus, demonstrating that antibiotic resistance can occur in a broad spectrum of pathogens causing pyelonephritis in pregnant women.

As expected, the bacteria identified in the study group showed a higher resistance to the antibiotics tested, especially to penicillins and cephalosporins, just as in other studies on MDR bacteria [34], which can be explained by the fact that urinary tract infections in pregnancy are usually treated with these antibiotics [35], so there is an increased risk of resistance. The high resistance to quinolones in the study group compared to the control group is noteworthy, although quinolones are not usually administered during pregnancy. This phenomenon could be due to the fact that some pregnant women may have received these antibiotics because of pre-pregnancy cystitis or pyelonephritis. However, although the MDR bacteria met this definition, they retained complete sensitivity to carbapenems and piperacillin/tazobactam, suggesting that in the presence of pyelonephritis in pregnant women who have risk factors for multidrug resistance, initiation of antibiotic therapy should begin with these antibiotics, especially in the case of associated sepsis [14].

The information presented in this article is of interest to urologists and gynecologists who are confronted with pyelonephritis in pregnant women, but also to infectious disease physicians to better know the particularities of MDR bacteria in pregnancy.

The main limitations of our study are the relatively small number of the study group, its retrospective nature and the absence of a multicentric study. Other limitations were selection bias, lack of generalizability, and the lack of multivariate analysis to account for confounding variables, given the small sample size and retrospective character of the study. However, considering the low incidence of this condition, and that such cases have not yet been reported in Europe, we believe that our study can make a useful contribution to the study of pyelonephritis in this particular group of patients.

## Conclusions

Pyelonephritis with MDR bacteria in pregnant women occurs in the second and third trimesters of pregnancy after antibiotic treatment and ureteral catheterization, predominantly on the right side. The clinical picture is dominated by the occurrence of urosepsis. The bacteria most frequently involved are E. coli and Klebsiella pn., which retain a sensitivity to carbapenems. Future prospective research on a larger cohort of patients is needed to validate our findings.
